# Coupling Additive Manufacturing with Hot Melt Extrusion Technologies to Validate a Ventilator-Associated Pneumonia Mouse Model

**DOI:** 10.3390/pharmaceutics13060772

**Published:** 2021-05-21

**Authors:** Bahaa Shaqour, Juliana Aizawa, Clara Guarch-Pérez, Żaneta Górecka, Lars Christophersen, Wim Martinet, Emilia Choińska, Martijn Riool, Bart Verleije, Koen Beyers, Claus Moser, Wojciech Święszkowski, Sebastian A. J. Zaat, Paul Cos

**Affiliations:** 1 Laboratory for Microbiology, Parasitology and Hygiene (LMPH), Faculty of Pharmaceutical, Biomedical and Veterinary Sciences, University of Antwerp, Universiteitsplein 1 S.7, 2610 Wilrijk, Belgium; juliana.aizawaportodeabreu@uantwerpen.be (J.A.); paul.cos@uantwerpen.be (P.C.); 2 Mechanical and Mechatronics Engineering Department, Faculty of Engineering & Information Technology, An-Najah National University, Nablus P.O. Box 7, Palestine; 3 Department of Medical Microbiology and Infection Prevention, Amsterdam Institute for Infection and Immunity, Amsterdam UMC, University of Amsterdam, 1105 AZ Amsterdam, The Netherlands; c.m.guarchperez@amsterdamumc.nl (C.G.-P.); m.riool@amsterdamumc.nl (M.R.); s.a.zaat@amsterdamumc.nl (S.A.J.Z.); 4 Faculty of Materials Sciences and Engineering, Warsaw University of Technology, Woloska 141, 02-507 Warsaw, Poland; zaneta.gorecka.dokt@pw.edu.pl (Ż.G.); emilia.choinska@pw.edu.pl (E.C.); wojciech.swieszkowski@pw.edu.pl (W.Ś.); 5 Centre for Advanced Materials and Technologies CEZAMAT, Warsaw University of Technology, Poleczki 19, 02-822 Warsaw, Poland; 6 Department for Clinical Microbiology, Rigshospitalet, Henrik Harpestrengsvej 4A, Afsnit 93.01, 2100 Copenhagen, Denmark; lars.christophersen@regionh.dk (L.C.); moser@dadlnet.dk (C.M.); 7 Laboratory of Physiopharmacology, University of Antwerp, Universiteitsplein 1 T.2, 2610 Wilrijk, Belgium; wim.martinet@uantwerpen.be; 8 Voxdale bv, Bijkhoevelaan 32, 2110 Wijnegem, Belgium; bart@voxdale.be (B.V.); koen@voxdale.be (K.B.)

**Keywords:** additive manufacturing, hot melt extrusion, extrusion die, extrusion nozzle, *Staphylococcus aureus*, antibiofilm, ventilator-associated pneumonia, ciprofloxacin, endotracheal tubes, anti-infective, medical devices, biomaterials

## Abstract

Additive manufacturing is widely used to produce highly complex structures. Moreover, this technology has proven its superiority in producing tools which can be used in different applications. We designed and produced an extrusion nozzle that allowed us to hot melt extrude drug-loaded tubes. The tubes were an essential part of a new mouse ventilator-associated pneumonia (VAP) model. Ciprofloxacin (CPX) was selected for its expected activity against the pathogen *Staphylococcus aureus* and ease of incorporation into thermoplastic polyurethane (TPU). TPU was selected as the carrier polymer for its biocompatibility and use in a variety of medical devices such as tubing and catheters. The effect of loading CPX within the TPU polymeric matrix and the physicochemical properties of the produced tubes were investigated. CPX showed good thermal stability and in vitro activity in preventing *S. aureus* biofilm formation after loading within the tube’s polymeric matrix. Moreover, the produced tubes showed anti-infective efficacy in vivo. The produced tubes, which were extruded via our novel nozzle, were vital for the validation of our mouse VAP model. This model can be adopted to investigate other antibacterial and antibiofilm compounds incorporated in polymeric tubes using hot melt extrusion.

## 1. Introduction

Hospitalized patients are at high risk of morbidity and even mortality from hospital-acquired infections (HAIs), in addition to their primary illness. According to the European Centre for Disease Prevention and Control, 8.9 million European patients acquire infections in hospitals and long-term care facilities every year [1]. HAIs cause over 90,000 deaths per year in European countries [2], more than any other infectious disease under surveillance. Hospital-acquired pneumonia is the second most common nosocomial infection and one of the most common causes of death from HAI in critically ill patients [3]. Ventilator-associated pneumonia (VAP) is a serious hospital-acquired pneumonia which is defined as lung infection acquired by patients mechanically ventilated in the intensive care unit for at least 48 h [4]. Patients with VAP have increased length of hospitalization and consequently increased healthcare cost [5].

Endotracheal tube microbial colonization is a major risk factor for VAP and a multispecies biofilm of commensal microorganisms forms in 90% of the tubes used in mechanical ventilation [6]. This biofilm presents a clinical challenge since pathogens can attach to the microbial community formed on the inner surface of the endotracheal tube. Carried by airway pressure, they can then bypass normal barriers and reach the pulmonary parenchyma or infect the lungs. In a prospective observational study that included patients from 27 European intensive care units (ICUs), *Staphylococcus aureus* is reported as the most common Gram-positive isolate in VAP patients and *Pseudomonas aeruginosa*, *Acinetobacter baumannii*, and *Escherichia coli* are among the most common Gram-negative isolates [7]. Mortality rates can vary in different patient groups and depend on underlying conditions and disease severity. In a meta-analysis using randomized trials and considering a homogeneous group of patients, the overall attributable mortality associated with VAP was 13% [8].

To address this high risk of mortality from VAP, several strategies have been investigated, such as modifying endotracheal tubes in order to make their surface antifouling and/or preventing biofilm formation by incorporating substances with antibacterial properties during the manufacturing process [9]. The limited number of available solutions is due to the complex process of developing reliable devices, starting from idea up to the market. For a successful development, an interdisciplinary approach is essential, requiring drug development, material science, engineering, clinically relevant animal models and, finally, well-designed clinical studies. Thus, the availability of tools to produce testing samples for each step of characterization and validation is key to achieving the required results. In this study, we focus on producing tubes for preclinical tests with a VAP mouse model.

The main manufacturing process used to produce tubing is hot melt extrusion. In this process, a thermoplastic polymer is melted and extruded through a specially designed nozzle (commonly known by the term “extrusion die” in the relevant literature) in the shape of the product [10]. Via this process, a wide range of products can be manufactured, from plastic profiles used in buildings, to tubes used for medical applications. Moreover, there are several examples of the latter application such as tubes and catheters being used in chemotherapy and neonates. The nozzles used in such processes are custom-made and composed of multiple components. An alternative approach to producing such nozzles can be via additive manufacturing (AM) technologies. These technologies have recently been used in the production of tools in different sectors [11,12]. They offer the unique possibility of layered manufacturing in producing complex objects in a cost- and time-effective manner. Moreover, AM has been used for the production of extrusion nozzles capable of extruding multiple materials with a coaxial complex design [13,14,15].

In this work, we propose a method for producing test tubes to validate a VAP mouse model via the outstanding capabilities of AM technologies. An extrusion nozzle was designed using computer-aided design software, and was 3D printed to be used as a tool for hot melt extruding tubes that can meet the demands of the mouse VAP model. A strict outer and inner diameter of 1.00 mm and 0.60 mm, respectively, is required to fit within the mouse trachea. The use of a mini single screw extrusion system was key to having a feasible system that allows testing different formulations. On the other hand, solvent casting was used to produce drug-loaded films. Ciprofloxacin (CPX), which is a conventional broad-spectrum antibiotic widely used to treat VAP infections, was chosen as an antibacterial and antibiofilm compound [16]. Thermoplastic polyurethane (TPU) was selected as the carrier polymer since it is widely used in many medical tubing applications [17].

The tubes were characterized for their thermophysical properties, release of CPX, and cytotoxicity. The antibacterial and antibiofilm properties of the tubes were assessed by antimicrobial activity testing, bacterial adherence evaluation, and scanning electron microscopy. The strain of choice was *S. aureus* ATCC 25923, an established biofilm-forming strain used in previous standardization studies and in the screening of new and repurposed drugs [18]. The insertion procedure of the tubes was based on a previously described *P. aeruginosa* model used to investigate treatment of VAP [19].

The main aim of the presented study is to establish a proof of concept for the development of a novel 3D-printed nozzle, which allows the extrusion of customized drug-loaded tubes which meet the demands of an *S. aureus* VAP mouse model. Our findings support the proposal that the manufacturing approach described can successfully produce CPX-loaded tubes that allow us to validate the developed VAP mouse model. Proof of concept is key for further use of this novel technology in the evaluation of VAP prevention by other promising antibacterial and antibiofilm compounds incorporated in hot melt extruded polymeric tubes.

## 2. Materials and Methods

### 2.1. Materials Used

The TPU used was Tecoflex EG-60D, Lubrizol, purchased from VELOX (Laren, The Netherlands). The polymer contains a soft segment and hard segment in a ratio of 3:1. It has a glass transition temperature of 23 °C, and a melting temperature of 63 °C. Its molecular weight is 7.89 kDa [20]. CPX was purchased from Acros Organics (Geel, Belgium). Some preliminary testing was performed using polylactic acid (PLA) filament which was purchased from 3d4makers (Haarlem, The Netherlands). It has a glass temperature around 50 °C and a melting range between 170 °C and 230 °C [21].

**Figure 1:**
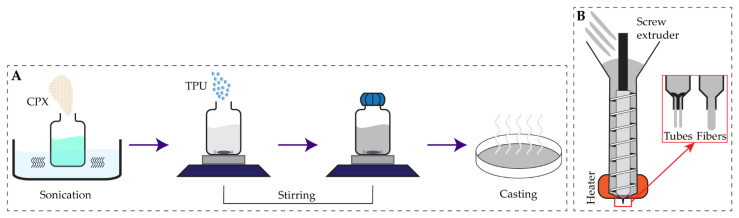
Solvent casting approach used to produce TPU and TPU-CPX films (**A**), hot melt extrusion process using a mini single screw extruder to produce fibers and tubes for material characterization and further in vitro and in vivo testing (**B**).

TPU was loaded with CPX using the solvent casting approach adapted from previous work by Shaqour et al. [22] (Figure 1A). To prepare the TPU-CPX film, 5% (*w/w*) of CPX was suspended in an organic solvent (chloroform) (Chempur, Piekary Śląskie, Poland) and sonicated for 30 min. Then, a magnetic stirrer (VELP Scientifica Srl, Usmate, Italy) was used for 8 h to homogeneously distribute the drug particles in the solvent. Afterwards, 95% (*w/w*) TPU was added to the system while stirring, and was left overnight to dissolve the polymer. The solution was then poured on a Petri dish (200 mm in diameter) to evaporate the solvent completely under the fume hood, resulting in solid TPU-CPX (95:5 *w/w*) films. Finally, the films were vacuum-dried for 3 days at 25 °C and 50 mbar. To prepare the TPU-only films, the same procedure was performed excluding the addition of CPX.

### 2.2. Nozzle Design and 3D Printing

**Figure 2:**
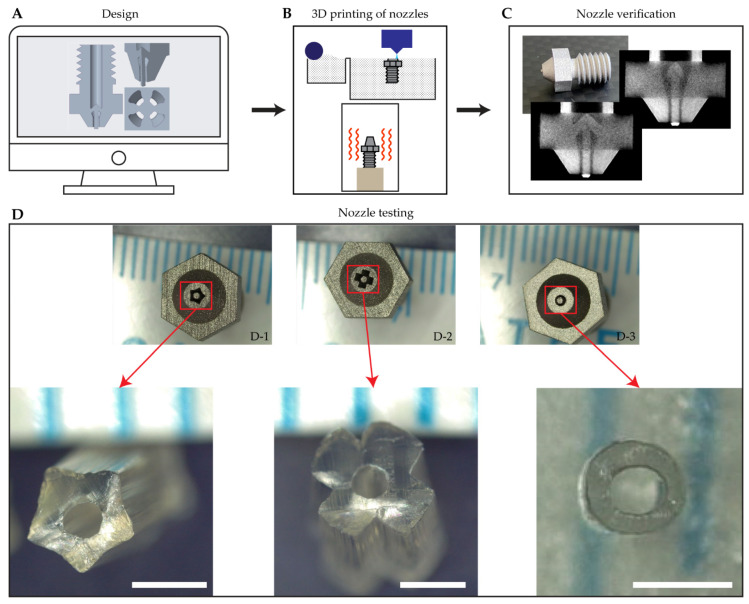
Methodology adopted to produce the extrusion nozzle used in the production of the tubes. Nozzle design (**A**), 3D printing of the nozzles (**B**), Nozzle’s internal structure verification (**C**), hot melt extrusion testing (**D**), scale bar represents 1 mm length.

A novel nozzle with the ability of extruding tubes was designed using Creo Parametric (v.5.0.4, PTC, Boston, MA, USA) (Figure 2A). Our nozzle was designed in a more simplified structure and taking into consideration the capabilities of AM technologies. The inlet of the nozzle has a diameter of 2.4 mm. The outlet of the nozzle has an outer diameter of 1 mm with a concentric coaxial rod with an outer diameter of 0.5 mm. The final design was sent to an external manufacturer (i.materialize, Leuven, Belgium). Metal 3D binder printing technology was used to produce the nozzle [23] (Figure 2B). In this technology, stainless steel fine powder is used as the raw material. A thin layer of powder is spread on top of the printing platform. Subsequently, the printing head deposits a binding material on selected parts of the layer in order to bind the metallic particles. Then, the printing platform is lowered, and the process is repeated. Finally, the objects produced in this process are placed in a sintering oven to decompose the binding material and fuse the metallic particles.

Microcomputer tomography scanning (μCT) measurements were performed using a SKYSCAN 1172 (Bruker, Kontich, Belgium) in order to check the internal geometry of the 3D-printed nozzles (Figure 2C). The scan of nozzles was performed at 100 kV and 100 μA over 180° with a rotation step of 0.42° and exposure time of 600 ms. A Cu+Al filter was used during the scanning process. The image pixel size was 7.95 μm. The obtained planar images were reconstructed and analyzed with the instrument software—NRecon and CTAn (v1.10.1.0, Bruker micro-CT, Kontich, Belgium).

### 2.3. Fibers and Tubes Extrusion

In order to test the ability of extruding tubes with complex cross section using the new nozzle design, several shapes were used (star, cross, and circular) in different nozzles (Figure 2(D1–D3), respectively). Preliminary testing for the 3D-printed nozzle was conducted using a PLA filament and extrusion temperature of 200 °C. This was done as PLA is widely available in the filament form and has suitable rheological properties when melted. The nozzle was fitted on a normal filament extruder usually used in fused filament fabrication 3D printers. Several extrusion experiments were conducted at different speeds.

TPU and TPU-CPX fibers and tubes were extruded using an in-house developed single screw extrusion set up (Figure 1B) attached to the designed coaxial nozzle (Figure 2(D3)) or an e3d v6 stainless steel nozzle (E3D-Online, Oxford, UK) with a 0.8 mm diameter. Verstraete et al. [20] conducted a screening for a wide variety of TPU grades and considered 180 °C suitable for processing the polymer. Thus, the extrusion was done at a temperature of 180 °C and the screw extrusion speed was set at 15 rpm. TPU and TPU-CPX films were cut into strips and manually fed into the extruder. The extruded fibers were cut into 40 mm pieces, while the extruded tubes were then cut into 10 mm pieces.

Tubes were inspected using a Leica s9i microscope (Leica, Machelen, Belgium). Pictures were analyzed using ImageJ software (v.1.52A, NIH, Bethesda, MD, USA) by calculating the equivalent diameter $\left(\sqrt{\frac{4\times Area}{\pi }}\right)$ of the measured area for the inner and outer circles for each tube (*n* = 6 for each group).

### 2.4. Mechanical Testing

In order to investigate the effect of CPX loading on the mechanical properties of the TPU polymer, tensile tests were performed on an AGS 5 kND tensile testing machine (Shimadzu, Duisburg, Germany). The tensile rate was set to 20 mm/min and the distance between grips was 20 mm. For simplicity, we used a simple geometry (fibers) to study the tensile properties of the CPX-loaded versus non-loaded TPU. Extruded fibers from a 0.8 mm nozzle were used for this test (*n* = 5 for each group). The tensile elastic modulus (EM) was calculated from the force-displacement curve generated by the machine’s software. A Matlab (v.R2020b, The Math-works, Inc., Natick, MA, USA) code was written to calculate the stress-strain curve and the slope of the best fit line between 50% and 150% strain.

### 2.5. Thermal Analysis

To study the thermal stability and the temperature of decomposition for the extruded materials, thermogravimetric analysis (TGA) was conducted using a Q5000 Analyzer (TA Instruments, New Castle, DE, USA). Tubes of around 10 mg were placed on a platinum pan and then a dynamic heating ramp of 20 °C/min with a resolution of 3.00 °C was performed up to 500 °C under a nitrogen flow of 60 mL/min.

Moreover, the thermal stability of the CPX was tested by heating its powder using the TGA machine for 5 min at 180 °C. Afterwards, non-heated and heated samples were compared to test whether the processing temperature affects the activity of the drug or not. The 5 min period was selected as it is close to but slightly longer than the expected residence time of the drug in the extruder.

### 2.6. Fourier-Transform Infrared Spectroscopy (FTIR)

CPX powder before and after heating to 180 °C was examined using FTIR spectroscopy in order to study the effect of heat on the drug’s molecular structure. Moreover, in order to study the interactions between the drug and the TPU molecules, FTIR analysis was conducted on the produced tubes. The FTIR spectrometer used (Nicolet 8700, ThermoScientific, Madison, WI, USA) is equipped with a diamond attenuated total reflectance (ATR) accessory. All ATR-FTIR spectra were recorded at room temperature in the 400–4000 cm^−1^ range. The spectral resolution and accuracy were 4 cm^−1^ and ±1 cm^−1^, respectively.

### 2.7. In Vitro Cell Proliferation Assay

A cell proliferation assay was performed on the CPX powder heated at 180 °C and not heated to evaluate the cytotoxic effects after heating. The L929 mouse fibroblast cell line was purchased from European Collection of Authenticated Cell Cultures (ECACC, Salisbury, UK) and cultured in cell culture flasks containing Dulbecco’s modified Eagle’s medium (DMEM, Thermofisher, Walthman, MA, USA) supplemented with an additional 10% of fetal bovine serum (FBS) (Thermofisher, Walthman, MA, USA) and 1% of penicillin-streptomycin (Thermofisher, Walthman, MA, USA). Cells were incubated at 37 °C, 5% CO_2_ and humidity. Cells were detached with trypsin-EDTA (0.25% trypsin, 1 mM EDTA, Thermofisher, Walthman, MA, USA) and counted for the seeding. Per well, 100 µL containing 1 × 10^3^ cells was seeded in a 96-well cell culture plate and cultured for 24 h at 37 °C and 5% CO_2_. The next day, the medium was removed and 200 µL of CPX (non-heated control or heated at 180 °C) at 10 µg/mL in DMEM was added. DMEM with 1% of penicillin and streptomycin was used as a positive control and DMSO at 10% was used as negative control. The cells were incubated for 24, 48, or 72 h at 37 °C and in 5% CO_2_. At each time point, the medium was removed, and cells were washed with 200 µL of phosphate buffered saline (PBS, Thermofisher, Walthman, MA, USA). To assess the percentage of viable cells, 100 µL DMEM and 20 µL of the reagent of Cell Titer 96 Aqueous One Solution Cell Proliferation Assay (Promega, Madison, WI, USA) were added to each well and incubated for 2 h at 37 °C and then the absorbance was measured at 490 nm.

### 2.8. In Vitro Antimicrobial Susceptibility Assay

To assess the minimal inhibitory concentration (MIC) and the minimal bactericidal concentration (MBC) of CPX, *S. aureus* ATCC 25923 was cultured to mid-logarithmic growth phase in tryptic soy broth (TSB) (Difco Laboratories Inc., New York, NY, USA) at 37 °C and 120 rpm (Heidolph Instrument, Schwabach, Germany) and diluted to approximately 1 × 10^6^ CFU/mL in TSB, based on the optical density of the suspension at 620 nm. From this bacterial inoculum, 10 μL was added to 90 µL of TSB containing CPX which had been heated at 180 °C for 5 min and cooled to room temperature, or non-heated CPX (control). CPX was serially diluted from 128 µg/mL to 0.125 µg/mL in TSB and cultured with the bacteria in 96-well plates with flat bottom (Greiner bio-one, Monroe, NC, USA) overnight at 37 °C, 120 rpm, in a box with a humidified atmosphere. As a control for bacterial growth, 10 µL of the inoculum was incubated in TSB without CPX. The MIC is defined as the lowest CPX concentration without visual growth. For the MBC, 2 × 10 µL of the undiluted samples, from the wells without visible growth and the well with the highest concentration that had visible growth (as a growth control), were plated on blood agar (Oxoid, Basingstoke, UK) and cultured at 37 °C overnight. The MBC is defined as the lowest CFX concentration that killed ≥99.9% of bacteria.

### 2.9. In Vitro Drug Release Assay

In order to measure the amount of CPX released from the extruded tubes both drug-loaded (*n* = 3) and non-loaded (*n* = 3), TPU tubes were cut into 3 mm length and weighted on a microbalance. Then, each tube was placed in a 1.5 mL Eppendorf tube (SafeSeal tube, Sarstedt Ag&Co, Nümbrecht, Germany) with 500 µL of PBS (Merck, PA, USA) and incubated at 37 °C and 120 rpm. The solution was changed at every time point (1 h, 6 h, 1 d, 2 d, 7 d, 14 d, 18 d, 24 d, 30 d, and 37 d). To measure the CPX concentration at each time point, 300 µL aliquots from each time point were placed in a well of a 96-well plate with flat bottom (Greiner bio-one, Monroe, NC, USA) and the absorbance at 330 nm was measured using a multi-well plate reader (Synergy H1, Biotek, Winooski, VT, USA). A calibration curve was plotted for CPX to estimate the concentration of the drug released from the tubes. This curve ranged from 1 to 50 µg/mL with *R*^2^ equal to 0.9998. The cumulative drug release was calculated based on the total loading amount (5% *w/w*) present in the tubes.

### 2.10. Agar Diffusion Assay

A modified Kirby–Bauer agar diffusion assay [24] was performed to determine the zone of inhibition (ZOI) of the 5% (*w/w*) CPX-loaded TPU and non-loaded TPU extruded tubes against *S. aureus* ATCC 25923. *S. aureus* was cultured to mid-logarithmic growth phase, in TSB at 37 °C and 120 rpm and diluted to 1 × 10^6^ CFU/mL. Two hundred microliters of the inoculum were spread on blood agar plates under sterile conditions. The 3 mm tube segments were inserted vertically in the inoculated agar plates in triplicates and the agar plates were incubated at 37 °C overnight. The next day, the tubes were transferred onto freshly inoculated blood agar plates; this step was repeated for 10 days. The resulting zones of growth inhibition were measured in mm.

### 2.11. In Vitro Bacterial Adhesion Assay

An in vitro bacterial adhesion assay was performed to quantify the numbers of bacteria that adhered to the extruded tubes. *S. aureus* was cultured to mid-logarithmic growth phase in TSB at 37 °C and 120 rpm and diluted in TSB to 1 × 10^6^ CFU/mL. The extruded tubes were incubated in 0.5 mL of this suspension in 1.5 mL Eppendorf tubes overnight at 37 °C and 120 rpm. The tubes were gently washed twice with demi water, placed in 1.5 mL Eppendorf tubes with 500 μL of PBS, vortexed 30 s and sonicated at 35 kHz for 15 min in a water bath sonicator (Elma Transsonic T460, Elma, Singen, Germany). This procedure does not affect the viability of the bacteria but releases them from the surface. The sonicates were serially diluted and the number of viable bacteria was determined by quantitative culture on blood agar plates.

### 2.12. Scanning Electron Microscopy (SEM)

SEM was performed to visualize the bacteria that adhered to the inside of the tubes. The set up was the same as in Section 2.11 until the 2 washing steps with demi water. Prior to SEM, samples were fixed in 4% (*v/v*) paraformaldehyde and 1% (*v/v*) glutaraldehyde (Merck, Kenilworth, NJ, USA) overnight at room temperature. Samples were rinsed twice with demi water for 10 min and dehydrated in a graded ethanol concentration series from 50% to 100% of ethanol. To reduce the sample surface tension, samples were immersed in hexamethyldisilazane (Polysciences Inc., Warrington, FL, USA) overnight and air-dried. Before imaging, samples were mounted on aluminum SEM stubs and sputter-coated with a 4 nm platinum–palladium layer using a Leica EM ACE600 sputter coater (Microsystems, Wetzlar, Germany). Images were acquired at 3 kV using a Zeiss Sigma 300 SEM (Zeiss, Oberkochen, Germany) at the Electron Microscopy Center Amsterdam (Amsterdam UMC, Amsterdam, the Netherlands). Of each tube, 8–10 fields were viewed and photographed at magnifications of 100× and 500×.

### 2.13. Preparation of the Pre-Incubated Tube and Determination of Bacterial Adhesion

After overnight growth, *S. aureus* ATCC 25923 cultures were centrifuged at 5251× *g* for 10 min and the resulting pellet was resuspended in 10 mL of TSB resulting in a suspension of approximately 1 × 10^8^ CFU/mL, the maximum start inoculum possible, in order to ensure successful establishing lung infection. The bacterial concentration was confirmed by viable plate count. Tube segments of 3 mm in length were incubated in 1 mL of this suspension for 4 h. The viability of the bacteria that adhered to the tubes was measured. For this assay, each tube was rinsed in TSB to remove planktonic bacteria and placed in 1 mL of sterile 0.5% Tween 20—TSB solution. For bacterial detachment, the tubes were vortexed for 20 s, sonicated for 5 min at 35 kHz at 20 °C, and vortexed for 20 s again. The number of viable bacteria was determined by quantitative culture of this sonicate.

### 2.14. Validation of the Anti-Infective Effect In Vivo

Twenty female SWISS-CD1 mice (Janvier Labs, Le Genest-Saint-Isle France) of approximately 25 g and 6 weeks old were kept in individually ventilated cages with HEPA filters as a barrier system, a constant temperature of 20–25 °C, average humidity of 50–60%, 12 h dark–light cycle, and food and drinking water ad libitum. Experiments were conducted when the mice were older than 12 weeks and approximately 45 g since at this age mice are considered adults [25]. This model was developed for *S. aureus* ATCC 25923 infection, since this bacterial strain is virulent enough to cause a chronic infection, without leading to extensive burden to the mice.

TPU tube segments were pre-incubated as described in the previous section and kept in an Eppendorf tube with 1 mL of PBS until insertion in the right main bronchus of the mouse by tracheotomy. The TPU-CPX tubes and TPU tubes were placed in the right main bronchus of the lungs, according to the protocol described by Yanagihara et al. [26] that has been used to study the treatment of *P. aeruginosa* endotracheal tube-related infection, with the addition of tracheotomy, as described by Facchini et al. [27].

**Figure 3:**
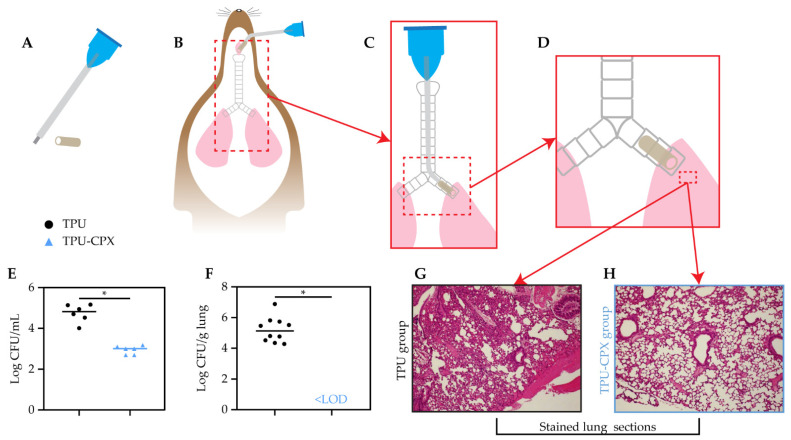
Protocol established for the *S. aureus* ATCC 25923 VAP mouse model (**A**–**D**). Log CFU/mL in the pre-inoculated tubes after 4 h incubation at 37 °C (**E**). The results are presented as the mean of 3 biological replicates with 4 technical replicates, each in 2 independent experiments, Log CFU/g in the left lung 3 days after insertion of the tubes in the right main bronchus. In the TPU group the bacterial burden was below the limit of detection (LOD) (**F**). Lung H&E stained sections of the TPU group (**G**) and TPU-CPX group (**H**), 10× magnification was used. Note: *n* = 6 per group in E, *n* = 10 per group in F, with 2 independent repeats and 5 animals in each. An unpaired *t*-test with Welch’s correction was performed and * indicates *p* < 0.05.

In brief, mice were anesthetized with 50 mg/mL ketamine (Nimatek 100 mg/mL, Dechtra Veterinary Products NV, Lille, Belgium) and 5 mg/mL xylazine (Rompum 2%, Bayer SA-NV, Machelem, Belgium) administered at 0.002 mL/g bodyweight in 0.9% saline solution by intraperitoneal injection. Then, the mice were placed in a supine position and the coat was disinfected with 70% ethanol. The trachea was exposed by a vertical cut of the skin and an opening was created in the exposed trachea with a 22G needle. Then, the tube was inserted through this opening with the help of a sterile, flexible 22G 0.9 mm × 25 mm intravenous catheter (Becton Dickinson Medical, Temse, Belgium) with a blunted needle and its outer sheath (Figure 3A) and the attached tube at the tip. The tube was advanced into the trachea until it reached the desired location in the right main bronchus. The inner needle was then pulled out, followed by a gentle push of the outer sheath to place the tube into the main bronchus (Figure 3B–D). The infected mice were observed for clinical signs (i.e., coat quality, posture, ambulation, and hydration) after surgery and had had unlimited access to feed and water. Body weight was monitored daily since mice that lose ≥20% bodyweight are considered in morbid status and must be euthanized.

After 3 days, the animals were euthanized by cervical dislocation, the left lung excised, weighed, and homogenized in 1 mL of PBS to determine the bacterial burden (CFU/g). For that, 100 μL of the homogenate was 10-fold serially diluted in PBS, and quadruplicate 10 µL droplets of each dilution were plated onto triple soy agar plates and incubated at 37 °C overnight. The bacterial burden was expressed per gram of lung tissue. The right lung was fixated in 4% paraformaldehyde for 24 h and kept in 60% isopropanol until the moment of processing for histology. Sections of murine lungs were stained with hematoxylin and eosin (H&E) and inflammation was scored by 2 independent observers at 400× magnification using an EVOS FL Auto microscope (Life Technologies, Belgium), according to Cigana et al. [28]. Briefly, neutrophil and macrophage counts classified the infection as absent (score of 0) when there were no or fewer than 19 cells per field, mild (score of 1) for 20 to 49 cells per field, moderate (score of 2) for 50 to 99 cells per field, and severe (score of 3) for 100 or more per field.

### 2.15. Statistical Analysis

Statistical analysis was performed with Graphpad prism 8 (GraphPad Software Inc., La Jolla, CA, USA.). Unpaired *t*-tests were performed in Section 2.11, Section 2.13 and Section 2.14. *** indicates *p* < 0.001 and * indicates *p* < 0.05.

## 3. Results

### 3.1. Nozzle Design, Production, and Preliminary Testing

The initial design was prepared using computer-aided design software. In this novel design, a nozzle with an inlet diameter of 2.4 mm and an outlet outer diameter of 1.00 mm with a concentric rod with a diameter of 0.5 mm was produced. The internal geometry of the nozzle includes a main channel (inlet) diverging into four subchannels in order to support the concentric rod (Figure 2A). Those channels converge into one main channel again with a rod aligned at the center. This setup allows the extrusion of tubes with outer diameter similar to the main channel and an inner lumen with a diameter similar to the concentric rod. However, the final dimension of the extruded tube will vary from the nozzle’s dimension due to polymer swelling [29].

After receiving the 3D-printed nozzles, μCT scanning was performed to check the internal structure. The nozzle was successfully 3D-printed with designed internal structure (Figure 2C). The concentric rod and its supports were printed very similarly to the original design. Moreover, the threading was produced in a good shape that allowed easy attachment of the nozzle to the extrusion system.

In order to test the potential of this approach, several designs were prepared for the nozzle to examine the ability of extruding star, cross, and circular tubes (Figure 2(D1–D3), respectively). The produced nozzles were attached to an extrusion head of a fused filament fabrication 3D printer. PLA filaments—a widely used material in 3D printing applications [30]—were used for this initial testing, resulting in extrudates with a shape similar to the nozzles. Moreover, the internal lumen was always preserved.

### 3.2. Production of TPU and TPU-CPX Fibers and Tubes

TPU was received in the form of pellets. The solvent casting approach (Figure 1A) was used to load the TPU with CPX via dissolving the polymer pellets in chloroform as an organic solvent. It was very important to select a preparation method that provides good homogeneity of the CPX distribution among the polymer matrix specially since the CPX solubility in organic solvents is very low [31] and CPX will mostly be suspended in the solvent. After evaporating the solvent, the films were examined for any possible agglomerates. The produced TPU films without CPX were transparent, and the TPU-CPX films were white with no observable agglomerates, indicating homogenous distribution of the CPX within the TPU matrix (Figure S1).

**Figure 4:**
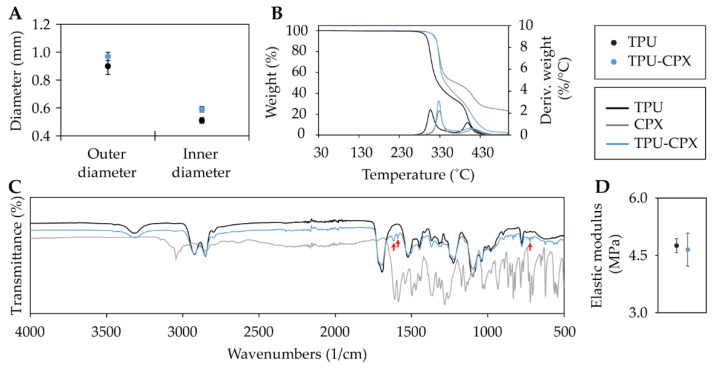
Outer and inner diameter of the extruded TPU and TPU-CPX tubes (**A**), thermogravimetric analysis for CPX, TPU, and TPU-CPX (**B**), FTIR spectra for CPX, TPU, and TPU-CPX, red arrows indicate CPX peaks (**C**), and tensile elastic modulus of TPU and TPU-CPX fibers (**D**). Note: in part B, Deriv. stands for derivative.

Films of TPU and TPU-CPX were prepared and then cut into strips and stored prior to extrusion. The produced strips were fed into the vertically positioned single screw extruder (Figure 1B). The extrusion temperature was set to 180 °C. A nozzle with outer diameter of 0.8 mm was used to extrude fibers used for the mechanical testing, while the newly designed nozzle for circular tubing was used to produce the tubes (Figure 2(D3)). The outer and inner diameters of the TPU tubes were 0.90 ± 0.06 mm and 0.51 ± 0.03 mm, respectively (Figure 4A), while the outer and inner diameters of the TPU-CPX tubes were 0.97 ± 0.02 mm and 0.59 ± 0.02 mm, respectively.

### 3.3. Thermophysical Properties of TPU and TPU-CPX Fibers and Tubes

TGA and FTIR analyses were used to test the thermal stability of the components for producing the tubes. TGA analysis showed that CPX powder, TPU, and TPU-CPX tubes had an onset of degradation temperature of 318 °C, 295 °C, and 321 °C, respectively (Figure 4B). The loading of CPX in the TPU matrix improved the polymers’ thermal stability by delaying the onset degradation temperature. Similar results were obtained by Mathew et al. [32] when loading TPU with tetracycline hydrochloride.

FTIR spectra of the CPX as received (i.e., non-heated) and of the heated sample at 180 °C for 5 min were compared (Figure S2). The heating appeared not to have caused observable changes in the spectrum. Additionally, the FTIR spectra of the TPU and TPU-CPX tubes and pristine CPX alone were compared (Figure 4C). In CPX spectra as well as in the TPU-CPX spectra, three distinctive peaks at 1610 cm^−1^, 1585 cm^−1^, and 620 cm^−1^ (red arrows: Figure 4C) were observed. This confirms the presence of the CPX particles within the TPU matrix. There are no large differences between the TPU and TPU-CPX spectra except for the peaks caused by the CPX which indicated that there was not much interaction between the TPU and the CPX molecules.

Additionally, tensile testing was conducted on the extruded fibers. The tensile EM was calculated from the stress-strain curve. The EM of the TPU and TPU-CPX fibers were 4.8 ± 0.2 MPa and 4.7 ± 0.4 MPa (Figure 4D). This indicates that there is no impact of the CPX loading on the mechanical properties.

### 3.4. Thermal Stability of the Drug and Cell Proliferation

CPX heated at 180 °C and non-heated CPX had the same MIC and MBC values (1 μg/mL), indicating that CPX is thermally stable at the processing temperature and should not lose its antimicrobial activity during the extrusion at 180 °C. Moreover, the cytotoxicity of CPX after heating was evaluated with the L929 mouse fibroblast cell line. We confirmed that the heating process does not generate cytotoxic compounds caused by any potential degradation of CPX since the activity of the treated cells ranged from 85 to 100%, indicating that there is no cytotoxic effect (Figure S3).

### 3.5. Drug Release, Antibacterial and Antibiofilm Activity of the TPU-CPX Tubes

**Figure 5:**
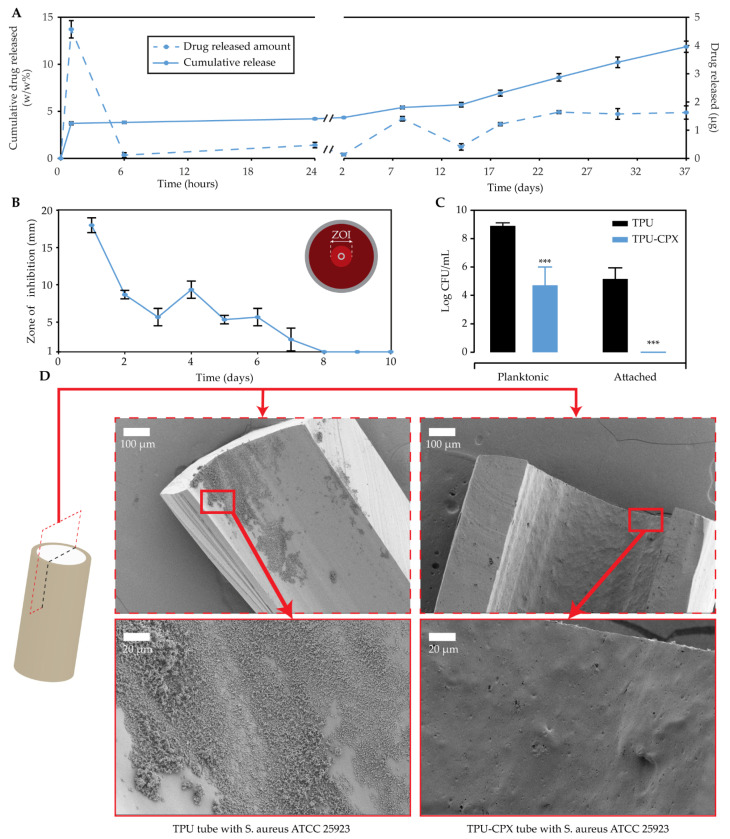
Released drug profile for TPU-CPX tubes: single timepoints (dashed) and cumulative (continuous) (**A**), bacterial zone of inhibition assay (**B**), bacterial adhesion assay: TPU (black) and TPU-CPX (blue) (**C**), and SEM visualization of bacterial attachment for cross-sections of non-loaded and drug-loaded TPU tubes with 100× and 500× magnification and 3 kV surface tension (**D**). Note: *n* = 3 in (**A**,**B**,**D**) and *n* = 6 in (**C**). An unpaired *t*-test was performed in C and *** indicates *p* < 0.001.

The TPU tube loaded with 5% CPX showed a burst release after 1 h (Figure 5A). This burst release can be attributed to the CPX particles located at the surface of the tubes. This was followed by a sustained release after 24 h for the following 37 days.

The antimicrobial activity of TPU and TPU-CPX tubes was evaluated overtime against *S. aureus* ATCC 25923 with a modified Kirby–Bauer assay for 10 days. The TPU-CPX tubes showed a zone of inhibition (ZOI) of an average of 17 mm the first day (Figure 5B), followed by an average ZOI of 8.6 mm and 5.6 mm the second and third day, respectively. Then, it increased to a ZOI of 9.3 mm the fourth day. The ZOI was maintained until day 7; however, from the 8th day onward, no ZOI was observed. These results confirm the presence of antimicrobial activity of the tubes for 7 days.

The TPU-CPX tubes completely prevented the attachment of *S. aureus* to the tubes after an incubation of 24 h in vitro, when compared to TPU tubes (Figure 5C). Moreover, a significant decrease in the numbers of CFU present in the medium was observed, owing to the CPX released. These results were visually confirmed with SEM (Figure 5D). In the inner part of the TPU tubes, a biofilm was formed. In contrast, there was no biofilm formation on the TPU-CPX tubes at all.

### 3.6. Biofilm Adherence and Validation of the Anti-Infective Effect In Vivo

The TPU-CPX tubes showed a 1.9 Log reduction of *S. aureus* ATCC 25923 in comparison with the TPU tubes after 4 h incubation in vitro (Figure 3E). More importantly, the TPU-CPX tubes prevented infection in vivo. The bacterial burden in the left lung of the mice that were intubated with TPU-CPX tubes was below the limit of detection of 1.4 CFU/g (corresponding to 25 CFU/mL, i.e., to one single CFU present in one of the four technical replicates), as opposed to the mean of 5.2 Log CFU/g found in the animals with TPU tubes, and there was no dissemination of the bacteria to liver and spleen (Figure 3F).

Histological analysis [28] showed higher inflammation in the tissue of the right lungs of the TPU group (score of 2; Figure 3G) as compared to the CPX-loaded TPU group (score of 0; Figure 3H).

## 4. Discussion and Conclusions

AM technologies are fundamental to produce an extrusion nozzle that allows us to hot melt extrude drug-loaded tubes to be used in a VAP mouse model. The production of micro tubing for medical applications has been intensively explored in the scientific community and the industry for preclinical use in small animal models and clinical use in chemotherapy and for neonates [33]. Typically, tubes are manufactured via hot melt extrusion techniques, allowing either single or multiple lumen(s) with outer diameters starting from 100 μm [33]. The nozzle designing process should take into consideration manufacturability with conventional processes and ease of assembly. It is pivotal to have a good understanding of the polymer’s melt behavior during extrusion, so that a tube with the required dimensions is produced [34]. Most extrusion systems are equipped with a channel to supply air within the lumen of the extruded tube, to avoid its collapse and adjust its final dimensions [35]. The manufacturability of extrusion nozzles with novel designs is still a limitation as subtractive technologies such as machining are mostly used. This implies the need for designing the nozzle in several parts that should be assembled during installation [36,37].

The advancement in AM technologies allows the production of highly complex designs with internal geometries that were not possible using conventional techniques. It permits the design and production of the nozzle as one single part and reduces the manufacturing cost. In our work, we have successfully produced a nozzle with a specially designed internal geometry for extruding tubes with a single lumen. Moreover, our design proves that an air stream within the tube’s lumen while extruding can be eliminated without loss of the internal cavity. Jin et al. [37] optimized the design of an extrusion nozzle based on a conventional design and manufacturing approach. Their nozzle assembly consisted of two parts: the nozzle (die) with an output diameter of 1.0 mm and a mandrel with an outer diameter of 0.7 mm. The mandrel section included a central hole with a diameter of 0.4 mm for supplying air flow to the inner cavity of the extruded tube during the extrusion process. When comparing their design with ours, the concentric rod in the middle of our nozzle’s output represents the mandrel part. Our nozzle has 1.0 mm and 0.5 mm outer and inner diameters, respectively, which represent the 1.0 mm and 0.7 mm ones in their design, respectively. In their experiments, an outer diameter that ranged between 0.6 mm and 1.6 mm and an inner diameter that ranged between 0.4 mm and 1.0 mm were achieved. The main technology that allowed these small diameters was the use of a pulling device. The purpose of this device was to pull the extruded tube before its total solidification. In our work, we produced tubes with an inner and outer diameter of 0.51 ± 0.03 mm and 0.90 ± 0.06 mm, respectively. These small dimensions, which were required for the VAP animal model, were possible without the need of a pulling device.

The aim of our study was to develop a test system for evaluating novel antimicrobial compounds in a VAP mouse model. Therefore, the tubes produced with this novel design were loaded with CPX, a well-known antibiotic for treating *S. aureus* VAP. The advantages of incorporating CPX in the tubes are its thermal stability and low cost. Additionally, incorporating CPX at 5% (*w/w*) did not alter the original mechanical properties of TPU fibers. Although fibers are solid and tubes are hollow, the fact that the stiffness of the bulk polymer in the fibers did not change, means that the overall stiffness of the tubes will likely not be affected by the CPX addition [38]. From a clinical perspective, CPX should not be used prophylactically, since it is the drug of choice for treatment of VAP in patients [16]. The tubes were not cytotoxic, and their antibacterial properties were demonstrated by zone of inhibition and quantification of the planktonic bacteria. Their antibiofilm properties were also confirmed by quantification and visualization of the attached bacteria.

Animal models of device-related infections can be divided into site-specific and subcutaneous models. The VAP mouse model is a site-specific model and allows the evaluation of the host response to endotracheal tubes located in the same position as in the clinic [39]. Here, AM technologies allowed us to produce test samples that fit in the main bronchus of the mouse. Other animal species have been used to study biofilm-related infections in endotracheal tubes, such as sheep, pigs, and dogs [40,41,42]. Mice have the advantages of their small size, relatively high reproductive rate, and the availability of antibodies, increasing the chance to bring new technologies and drugs from bench to bedside. We used fully mature adult mice to ensure that the tubes fit in the trachea. Outbred SWISS-CD1 mice are used in all areas of biomedical research and are more resistant to infections than inbred mice as reported in a *P. aeruginosa* VAP model [25].

*S. aureus* is the most common cause of VAP among Gram-positive bacteria [7,43] and the PRINT-AID consortium has identified antibiofilm leads against *S. aureus* that could be incorporated into endotracheal tubes [44]. The challenge to establish a VAP mouse model lies not only in the technical, but also in the biological aspects. The selection of a bacterial strain is fundamental for the success of murine models [39]. The benefit of our animal model is the relative ease to establish a chronic infection (≥3 days) [45] with *S. aureus* ATCC 25923. The limitation is that the delivery of bacteria at implantation of the device is initially different between the CPX and the TPU tubes, since the bacterial viable numbers are reduced during the pre-incubation step due to eluting CPX. However, in the clinical situation an endotracheal tube is placed in the trachea in contact with the fluid lining of the tracheal epithelium. In this wet environment, CPX elution will also start at the same time as the first contaminating bacteria will reach the tube. Thus, it might be argued that the exposure of the bacteria to CPX in the pre-incubation step represents the situation that may occur in vivo. In future studies, it would be interesting to focus on pathophysiology by inserting non-colonized tubes and subsequently exposing mice to the bacteria. Other bacterial strains and even species could be used for this purpose, such as *P. aeruginosa,* since chronicity of infection has been previously obtained with the appropriate strain and methodology [26,46].

Although CPX-loaded tubes will not be used in the clinic, they were highly functional to validate our in vivo VAP model. The tubes were successfully manufactured using the hot melt extrusion process and the novel nozzle. The developed approach can be adopted for investigating other promising antibacterial and antibiofilm compounds and formulations incorporated in experimental endotracheal tubes. Moreover, our newly developed nozzle may be used for producing micro tubing in the medical and veterinary fields.

## Data Availability

Not applicable.

## Supplementary Materials

The following are available online at https://www.mdpi.com/article/10.3390/pharmaceutics13060772/s1, Figure S1. Produced TPU and CPX-TPU films after solvent casting, Figure S2. FTIR spectra of CPX as received (blue line) and heated for 5 min at 180°C (orange line), Figure S3. Percentage of L929 mouse fibroblast cell survival assay used to confirm that the heated CPX at 180 °C is not cytotoxic at 24, 48 and 72 h. Note: DMEM medium is the positive control and it is set at 100% cell survival and 10% DMSO is the negative control (*n* = 6).
