# Supplementary material for: Coupling Additive Manufacturing with Hot Melt Extrusion Technologies to Validate a Ventilator-Associated Pneumonia Mouse Model
Source: Pharmaceutics. 2021 May 21;13(6):772. doi: 10.3390/pharmaceutics13060772 (PMC8224298; doi:10.3390/pharmaceutics13060772)
Supplement: Supplementary file 1 [file pharmaceutics-13-00772-s001.zip › pharmaceutics-1184682-supplementary.pdf]

pharmaceutics

MDPI

Supplementary Materials: Coupling Additive Manufacturing with Hot Melt Extrusion Technologies to Validate a Ventilator-Associated Pneumonia Mouse Model

Bahaa Shaqour, Juliana Aizawa, Clara Guarch-Pérez, Żaneta Górecka, Lars Christophersen, Wim Martinet, Emilia Choińska, Martijn Riool, Bart Verleije, Koen Beyers, Claus Moser, Wojciech Święszkowski, Sebastian A. J. Zaat and Paul Cos

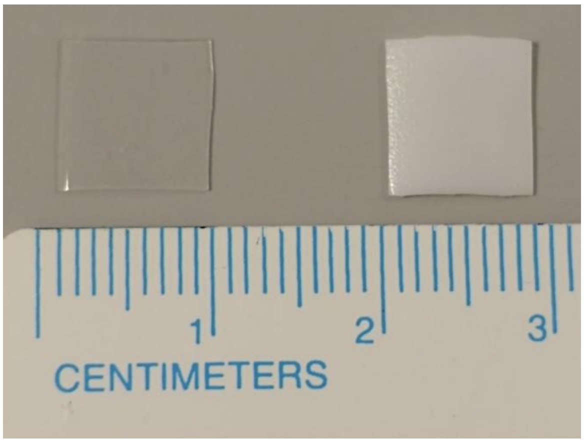

Figure S1. Produced TPU and CPX-TPU films after solvent casting.

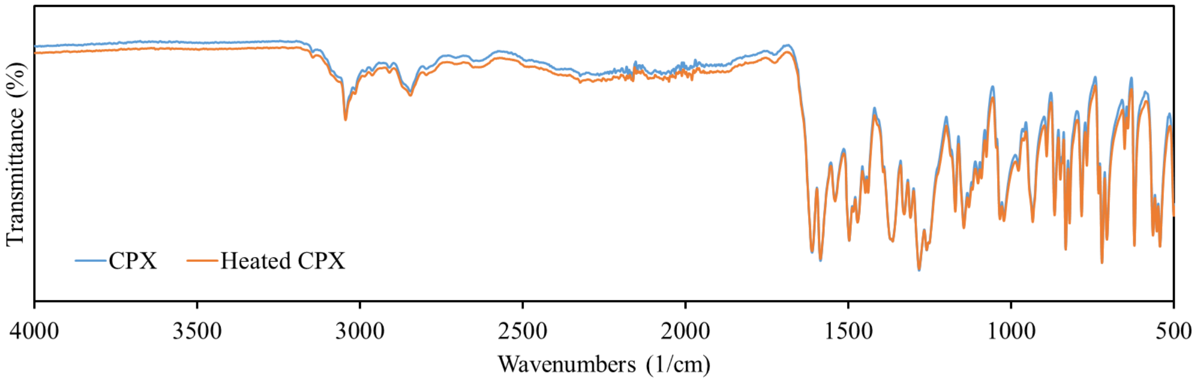

Figure S2. FTIR spectra of CPX as received (blue line) and heated for 5 min at 180 °C(orange line).

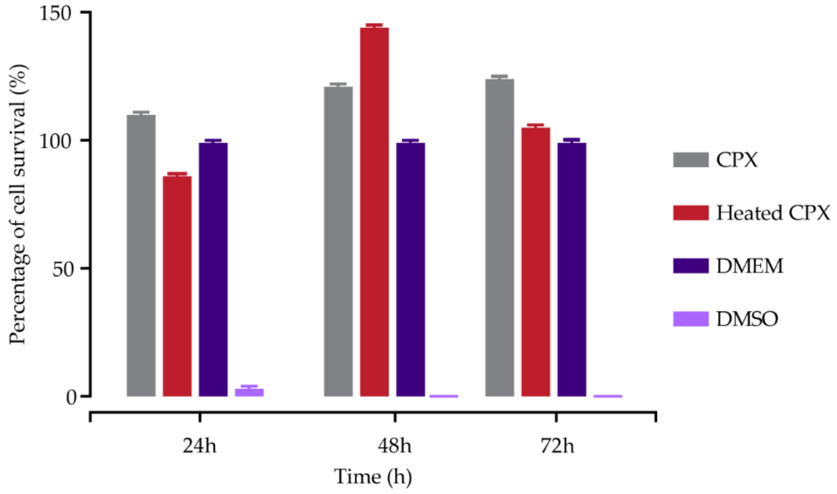

Figure S3. Percentage of L929 mouse fibroblast cell survival assay used to confirm that the heated CPX at 180 °C is not cytotoxic at 24, 48 and 72 h. Note: DMEM medium is the positive control and it is set at 100 % cell survival and 10 % DMSO is the negative control (n = 6).
